# 

**Published:** 1909-12

**Authors:** 


MARRIED.—Mabel, the charming daughter of Dr. and Mrs. J. H. Evans, of Palestine, was married October 26th to Mr. J. M. Norsworthy, of that city.

